# Supplementary material for: Structural and Functional Characterization of Rv0792c from Mycobacterium tuberculosis: Identifying Small Molecule Inhibitor against HutC Protein
Source: Microbiol Spectr. 2022 Dec 12;11(1):e01973-22. doi: 10.1128/spectrum.01973-22 (PMC9927256; doi:10.1128/spectrum.01973-22)
Supplement: Supplemental file 2 — Supplemental material. Download spectrum.01973-22-s0002.pdf, PDF file, 1.7 MB [file spectrum.01973-22-s0002.pdf]

Supplementary Information

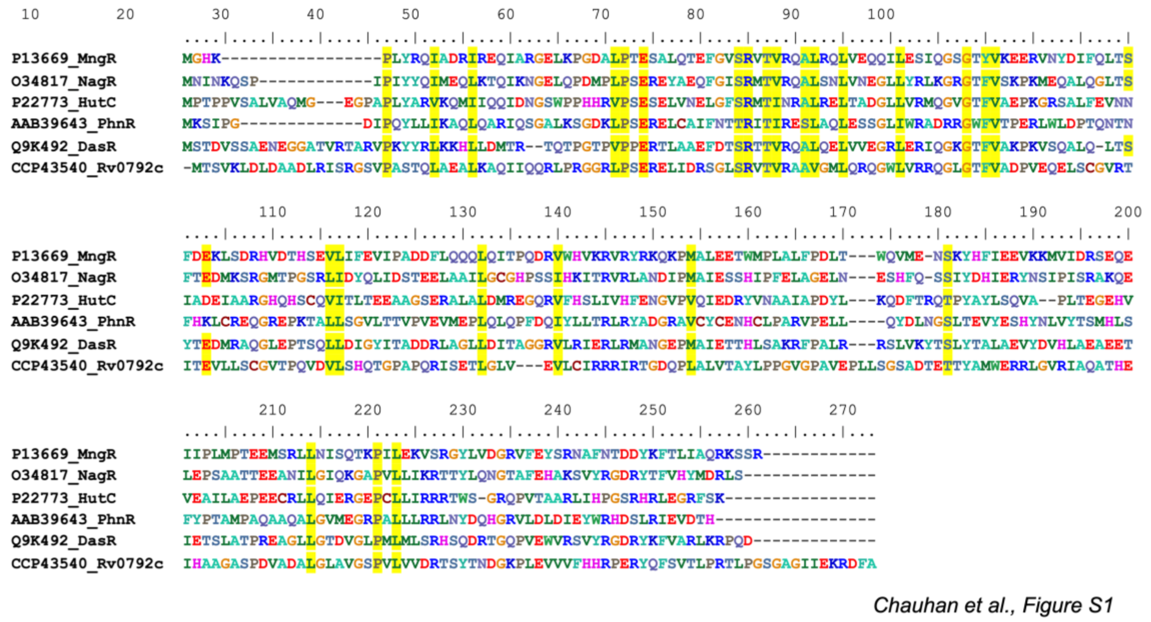

Figure S1: Multiple sequence alignment of putative Rv0792c homologs from various bacterial pathogens. Multiple sequence alignments among different proteins were prepared using Clustal W and formatted using BioEdit sequence alignment editor. The conserved residues among different proteins are highlighted in red. The proteins used in alignment are; CCP43540 (Rv0792c, *M. tuberculosis*), P13669 (MngR, *E. coli*), O34817 (NagR, *B. subtilis*), P22773 (HutC, *P. putida*), AAB39463 (PhnR, *S. enterica*) and Q9K492 (DasR, *S. coelicolor*).

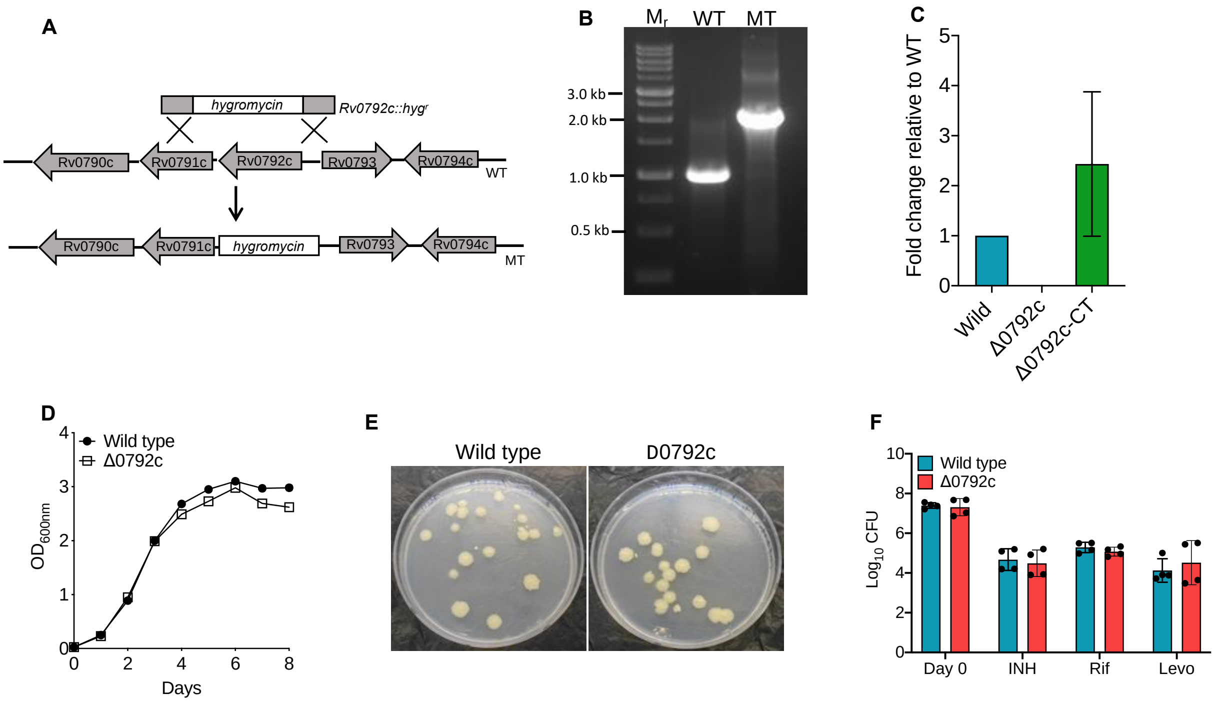

Chauhan et al., Figure S2

Figure S2: (A) Schematic representation of Rv0792c locus in wild type and mutant strain of *M. tuberculosis.* The gene for Rv0792c was replaced with the hygromycin resistance cassette in the mutant strain by homologous recombination using temperature sensitive mycobacteriophages **(B-F) Characterization of Rv0792c mutant strain of *M. tuberculosis***. The disruption of Rv0792c in the mutant strain was confirmed by PCR (B) and qPCR analysis (C) using gene locus specific primers. (D) The growth kinetics of wild type and Rv0792c mutant strain was compared by measuring the absorbance at 600nm. (E) The colony morphology of wild type vs mutant strain is shown in this panel. The data shown in panel D and E is representative of two independent experiments. (F) For *in vitro* drug susceptibility assays, mid-log phase cultures of various strains were exposed to various TB drugs for 14 days. The data shown in this panel is mean $\pm$ S.D. of results obtained from two independent experiments performed in duplicates.

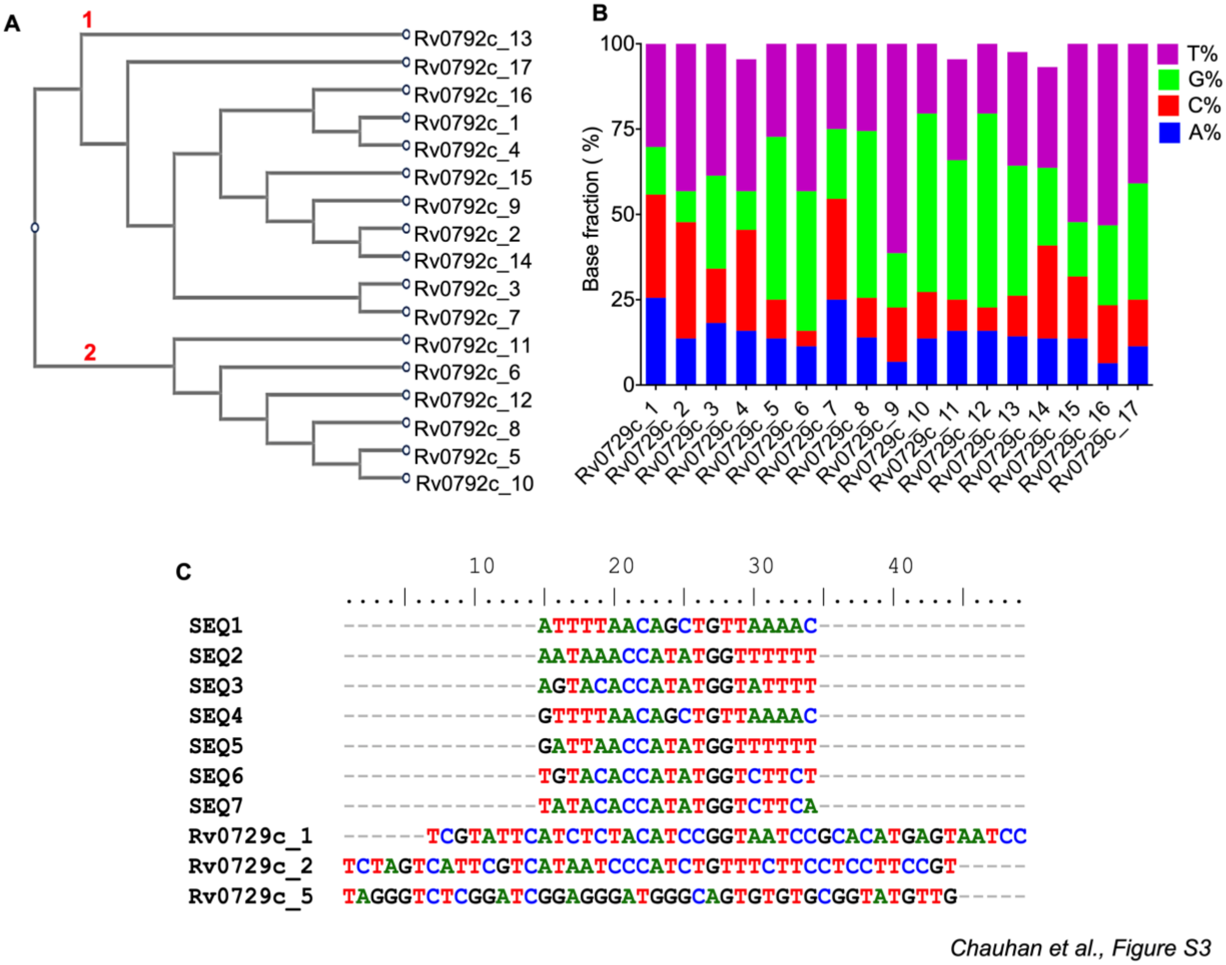

Figure S3: (A) Phylogenetic tree analysis of aptamer sequences using online tool ClustalW. The numbers in red denote the preponderant groups in the phylogenetic tree. **(B)** Base fraction analysis of aptamer candidates. We observed that the majority of selected aptamers evinced bias towards 'T' richness. **(C)** Multiple sequence alignment of Rv0792c_1, Rv0792c_2 and Rv0792c_5 aptamers with the known DNA sequences having affinity for transcription factors of GntR family. Seq1 to Seq7 represent the known DNA sequences.

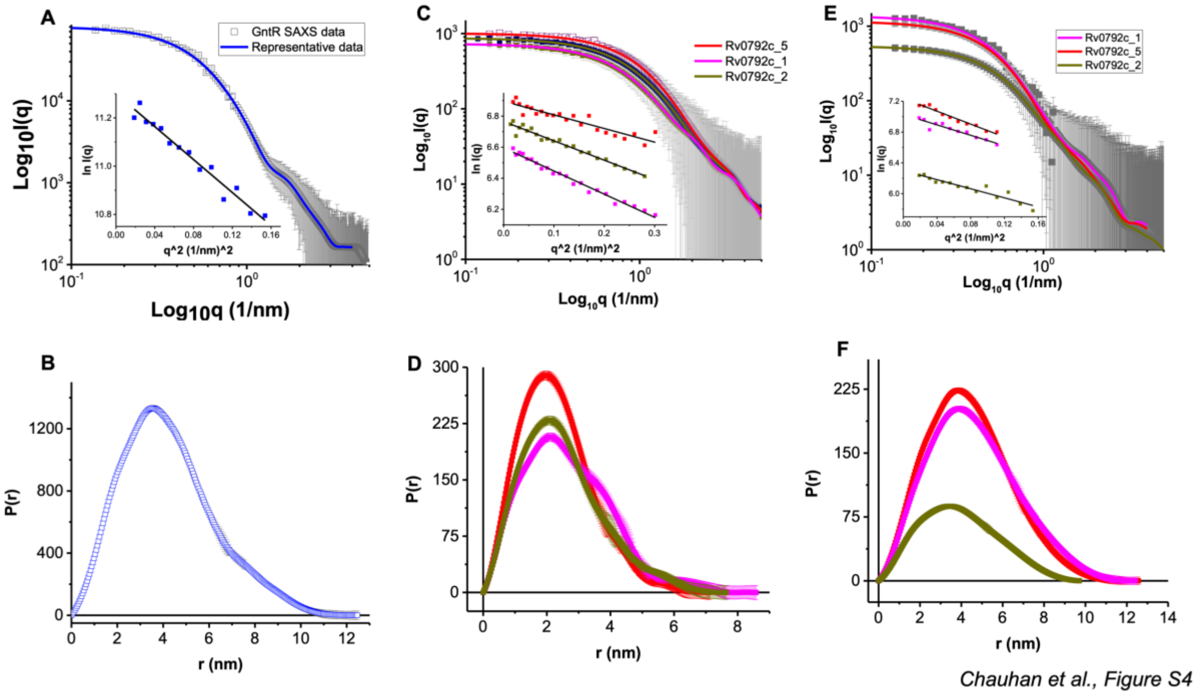

Figure S4: The panel **(A)** shows SAXS data acquired from a sample of Rv0792c at concentration close to 3.2 mg/ml (grey square symbols). Inset shows the linear region of the SAXS dataset in Guinier analysis considering globular scattering profile. **(B)** This panel shows the distance distribution profile of the interatomic vectors inside SAXS profile of the protein, and the blue line in the left panel plot shows the SAXS profile of the estimate. **(C-F)** Same analyses of the unliganded aptamers (**C, D**) and 1:1 molar mixture of protein and aptamers (**E, F**) are shown, respectively.

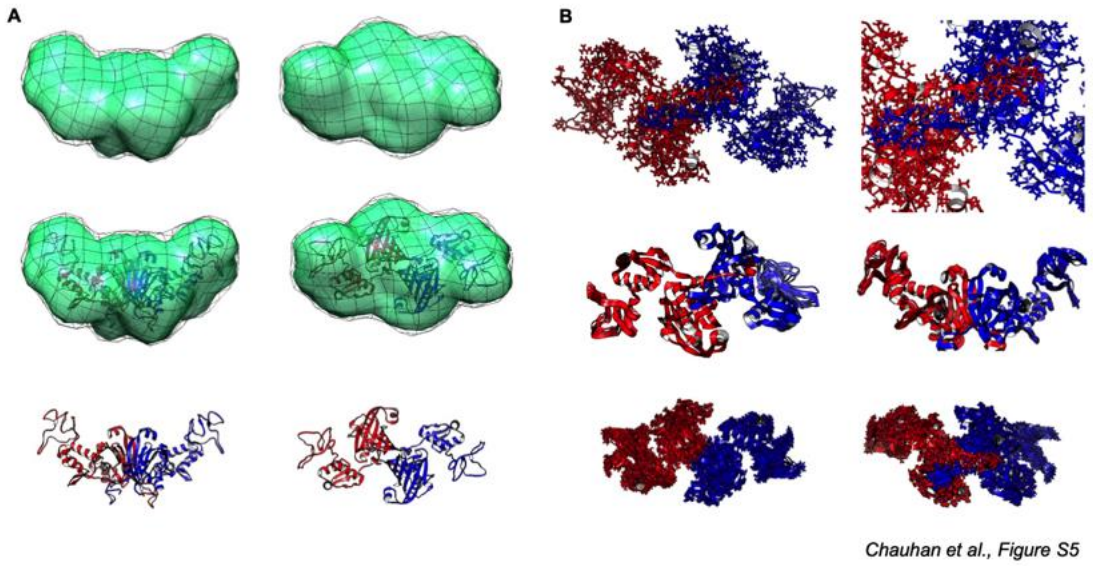

Figure S5: (A) Two orthogonal views compare the SAXS data based scattering envelope shape of Rv0792c and its structure solved by a combination of homology modelling and energy minimization. The green map represents the shape profile which was calculated to be common in ten dummy residue models of the protein. The variation is depicted as black mesh. The bottom panels show rotated views of the residue detail model of protein which was found to be a dimer and is shown in ribbon format. The central panel shows the inertial axes overlay of the two models indicating their similarity in three-dimensional space. **(B)** The calculated low frequency normal modes of motion accessible to the model of the Rv0792c dimer. Upper two panels highlight how the C-terminal tail of one chain latches on to the other to stabilize dimeric association. Lower four panels show calculated collective motion in the protein structure which shows motion in the N-termini of the protein chains in the dimer.

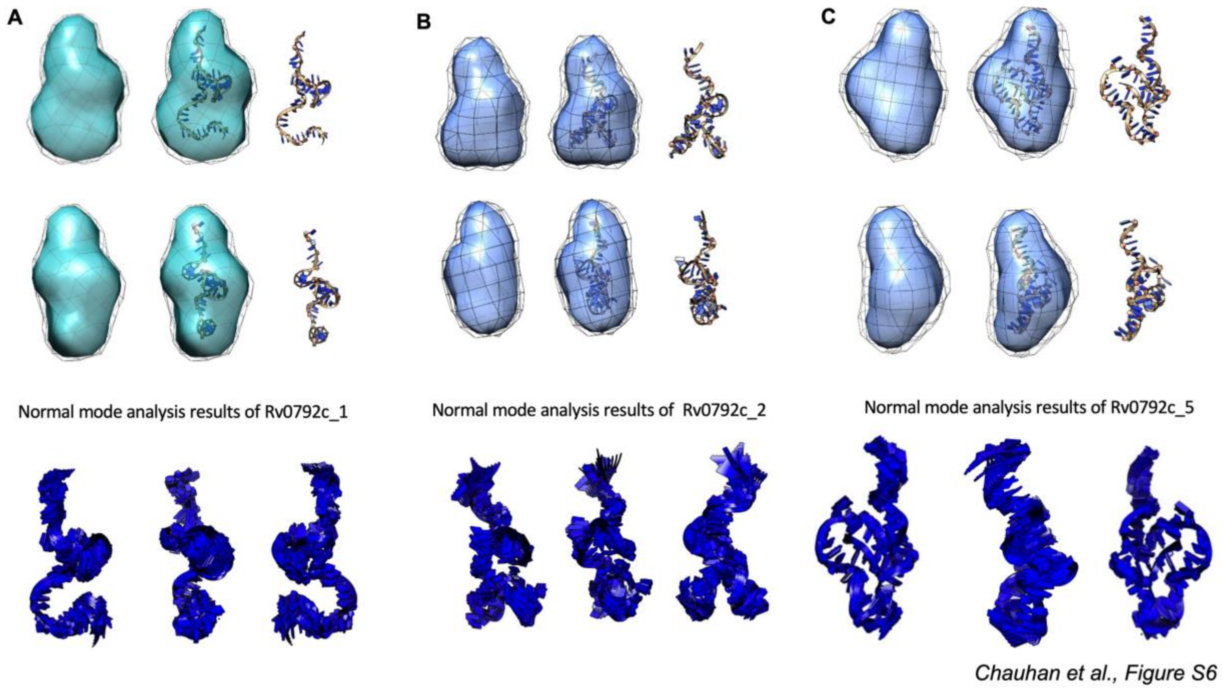

Figure S6: The SAXS based shapes of the three ssDNA aptamers have been shown here. The maps indicate the shape common to ten independent dummy residue solutions and black mesh represents the variation in them. The right panels show the low energy conformation of aptamer which best resembled the shape profile obtained experimentally in their SAXS profiles. All aptamers are monomer in solution. Normal mode analysis of the residue detail models of the aptamers have been done to perceive the extent of inherent motion accessible to the model of aptamers which also explains to some extent the additional volume seen in the molecular map of scattering shape of aptamers.

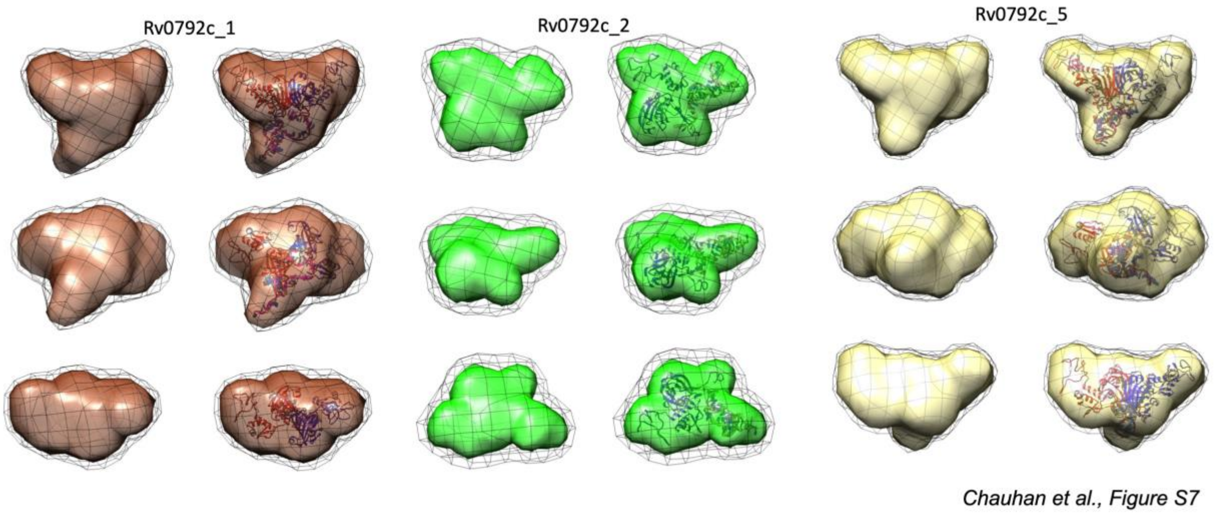

Figure S7: The SAXS based envelope shapes and residue scale models of the three ssDNA aptamers bound with Rv0792c protein have been shown here. The maps indicate the shape common to ten independent dummy residue solutions and black mesh represents the variation in them. The left column of images for the three sets shows the envelope of the complexes. The right column has the residue detailed model of the protein and aptamer where the latter was docked on protein and its pose was selected based on agreement with experimental SAXS data profile. The residue detail model was superimposed on the SAXS data based envelope in automated manner by aligning their inertial axes.

Table S1: List of strains, plasmids and primers used in the study.

Strains	Description	Source
$H_{37}Rv$	Laboratory strain (ATCC 27294) of *M. tuberculosis*	ATCC
Δ0792c strain	Rv0792c mutant strain of *M. tuberculosis*	This work
Δ0792c-CT strain	Rv0792c mutant *M. tuberculosis* strain complemented with Rv0792c	This work
BL21-CodonPLus (DE3)	F^{-} *ompT hsdS*(r_B^{-} m_B^{-}) *dcm*$^{+}$ Tet^{r} *gal* λ(DE3) *end*A Hte [*argU ileY* BB *leuW* Cam^{r}]	Stratagene, USA
E. coli HB101	F^{-}, *thi*-1, *hsdS*20 (r_B^{-} m_B^{-}), *supE*44, *recA*13, *ara*-14, *leuB*6, *proA*2, *lacY*1, *galK*2, *rpsL*20 (str^{r}), *xyl*-5, *mtl*-1	Promega, UK
E. coli XL-1 blue	*recA*1, *endA*1, *gyrA*96, *thi*-1, *hsdR*17, *supE*44, *relA*1, *lac* [F´ *pro*AB, *lacI*^{q}ZΔ*M15* Tn*10* (Tet^{r})]	Stratagene, USA
E. coli DH5α	*$F^{–}$ φ80lacZΔM15 Δ(lacZYAargF)U169 recA1 endA1 hsdR17($r_K^{–}$, m_K^{+}) phoA supE44 $λ^{–}$ thi-1 gyrA96 relA1*	Thermo Fischer, Scientific, USA
**Plasmids**	**Description**	**Source**
pGEM-T easy	T/A cloning vector, *amp*^{r}	Promega, UK
pTZ57 R/T	T/A cloning vector, *amp*^{r}	Thermo Fischer Scientific, USA
pYUB854	Cloning vector, *hyg*^{r}	Bardarov *et al*., 2002
pYUB854Δ0792c	pYUB854 vector containing upstream and downstream regions of Rv0792c, *hyg*^{r}	This work
pYUB159	Phagemid DNA, *amp*^{r}, temperature sensitive mycobacteriophage	Bardarov *et al*., 2002
pYUB159-Δ0792c	Phagemid DNA containing upstream and downstream region of Rv0792c, *hyg*^{r}	This work
pMV306K	*E. coli* mycobacterium shuttle vector, *kan*^{r}	Stover *et al*., 1991
pMV306K-0792c	pMV306K carrying Rv0792c along with its upstream region	This work
pET28b	*E. coli* T7 based expression system, *kan*^{r}	Novagen
pET28b-0792c WT	pET28b carrying Rv0792c coding region	This work
pET28b-0792c $_{R49A}$	pET28b carrying Rv0792c with Arg49-Ala mutation	This work
pET28b-0792c $_{G80D}$	pET28b carrying Rv0792c with Gly80-Asp mutation	This work
pET28b-0792c $_{R41A}$	pET28b carrying Rv0792c with Arg41-Ala mutation	This work
pET28b-0792c $_{P40A}$	pET28b carrying Rv0792c with Pro40-Ala mutation	This work

**Primers name**	**Forward (5’----- 3’)**	**Reverse (5’ ------- 3’)**
Rv0792c_up	GAGGCCTCGCACAGGCTGGTCACCGGATC	GTCTAGAAGCGTCCAGGTCCAGCTTGACAG
Rv0792c_dn	GAAGCTTCGAGATTTCGCATGAACGCCAAAG	GACTAGTCAGCAGCGTGACCGACAGTCGC
Rv0792c_ORF	GGGATCCGATGACATCTGTCAAGCTGGACCT	GAAGCTTTCATGCGAAATCTCGTTTCTCGATA
Rv0792c_R49A	TTGCCCAGCGAAGCAGAATTGATCGAC	GTCGATCAATTCTGCTTCGCTGGGCAA
Rv0792c_G80D	GTGCGCCGGCAAGACTTGGGTACCTTC	GAAGGTACCCAAGTCTTGCCGGCGCAC
Rv0792c_R41A	CAGCAGCGGCTGCCGGCCGGCGGGCGC	GCGCCCGCCGGCCGGCAGCCGCTGCTG
Rv0792c_P40A	CAGCAGCGGCTGGCGCGCGGCGGGCGC	GCGCCCGCCGCGCGCCAGCCGCTGCTG
Rv0792c complemented	GTCTAGAGCTGGTATAGCCAACCCGCCGCCG	GAAGCTTTCATGCGAAATCTCGTTTCTCGATA
Rv0792c_SYBR	GTGCGCCGGCAAGGCTTGGGTAC	GCGCCGGCGAATACAGAGGACC
Rv0792c promoter_Cy5	GTCTAGAGCTGGTATAGCCAACCCGCCGCCG	GGATATCAAGACGTATTAAAACGTCTTATACTCACCACG

Table S2a:
Details of instrumentation, programs used for SAXS processing and SAXS data based parameters of unliganded protein Rv0792c, aptamers found to bind the protein and their molar mixtures are tabulated below.

**Instrument**	**SAXSpace (Anton Paar, Austria)**
Collimation	Line Collimation
Source	X-rays, CuKα, 0.15414 nm
Detector	1D Mythen
Sample to detector distance	317.06 mm
Exposure time & Repeats	Average of three 60 minutes for samples & buffer
Subtraction from Solutions	Matched Buffer
**Programs**	
Data collection & Optics Control	SAXSDrive
Beam Position Correction	SAXSTreat
Buffer Subtraction & Desmearing	SAXSQuant
SAXS Intensity File Analysis	ATSAS Suite of Programs v 2.8.4
**Programs for Shape Restoration**	
Guinier Analysis	SAXS Data Analysis
Distance Distribution Function	SAXS Data Analysis
Shape Restoration	DAMMIF (10 independent runs, no symmetry bias)
Averaging	DAMAVER
Refinement of average	DAMMIN
Superimposition	SUPCOMB [Plugins for PyMol v 1.1]
Theoretical SAXS Comparison	CRYSOL
**Model Image Generation**	
Software	UCSF CHIMERA v 1.14

Table S2b

SAXS Data Analysis and Shape Reconstruction Related Parameters

Sample	*Guinier Analysis*	*Distance Distribution Function*		*NSD (Models)*	χ^2 *to residue model*
	R_g (nm)	D_{max} (nm)	R_g(nm)		
Unliganded Protein					
GntR	3.30	12.5	3.31	0.93±0.2 (10)	1.3
Unliganded Aptamers					
Rv0792c_5	1.81	7.1	1.84	0.549±0.02 (10)	1.2
Rv0792c_1	2.10	8.6	2.23	0.727±0.03 (10)	1.7
Rv0792c_2	1.92	7.6	2.01	0.684±0.02 (10)	1.5
1:1 molar mixtures					
P+ Rv0792c_5	3.33	12.5	3.40	0.92±0.1 (10)	1.7
P+ Rv0792c_1	3.52	12.4	3.55	0.84±0.2 (10)	1.8
P+ Rv0792c_2	3.10	9.8	3.08	0.78±0.1 (10)	1

Table S4:: Web-based tool MEME-Motif discovery predicted motifs among aptamer sequences

Motif	E-value	% Occurrence	Sites
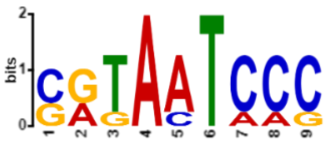	2.5e+000	29.41	**Rv0729c_2 AGTCATTCGT CATAATCCC ATCTGTTTCT**<br>**Rv0729c_3 TCGGGTGCT CGTAATCAC TTGAATGAGT**<br>**Rv0729c_14 GGTCTTATTG CGTACTCCC GGTTCCA**<br>**Rv0729c_1 CTCTACATCC GGTAATCCG CACATGAGTA**<br>**Rv0729c_7 ACAGTTCCTC GAGAATACC ACTGACTATT**
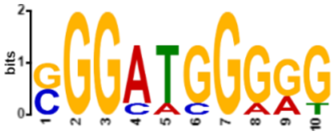	4.8e+001	35.29	**Rv0729c_8 TTGGATGGGT CGGATGGGGG**<br>**Rv0729c_13 TCG GGGATGGGGG GGGATGTTCT**<br>**Rv0729c_5 TAGGGTCT CGGATCGGAG GGATGGGCAG**<br>**Rv0729c_11 TAG GGGATGGAGG GTAATGTGGT**<br>**Rv0729c_1 GCATGCACTG GGGCTGGGAG CGCGGATGTT**<br>**Rv0729c_12TGTATGGGTC CGGAAGGGGT G**
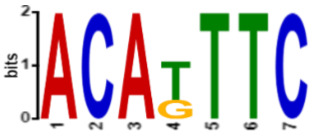	2.1e+002	17.64	**Rv0729c_16 TTTTTTCTGG ACATTTC GTGGGCCTT**<br>**Rv0729c_4 GGTTTTCAAT ACATTTC GATNCCCG**<br>**Rv0729c_7 TCGTTG ACAGTTC CTCGAGAATA**
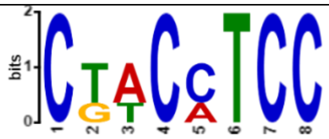	4.8e+002	17.64	**Rv0729c_4 T CGACCTCC TTTNTCTCTA**<br>**Rv0729c_1 GTATTCATCT CTACATCC GGTAATCCGC**<br>**Rv0729c_2 CCATCTGTTT CTTCCTCC TTCCGT**
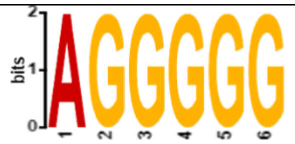	8.2e+002	23.52	**Rv0729c_12 T AGGGGG AGGAGCGGGT**<br>**Rv0729c_10 T AGGGGG TGGTGCATGC**<br>**Rv0729c_8 T AGGGGG GTTCCTTTCA**<br>**Rv0729c_6 GATGTGGTAG AGGGGG GTGGGGTTCT**
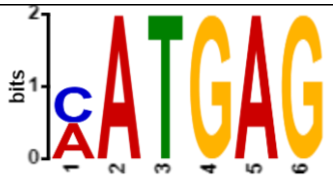	1.1e+003	11.76	**Rv0729c_3 TAATCACTTG AATGAG TTTATTGTGT**<br>**Rv0729c_1 GTAATCCGCA CATGAG TAATCC**
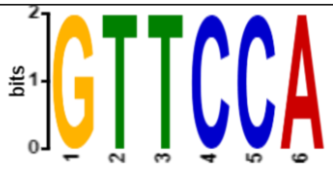	1.4e+003	11.76	**Rv0729c_15 TCGTC GTTCCA TCGTAAATTA**<br>**Rv0729c_14 CGTACTCCCG GTTCCA**
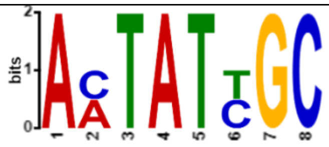	1.5e+003	11.76	**Rv0729c_17 TTTGCCGGTT AATATCGC**<br>**Rv0729c_7 AATACCACTG ACTATTGC AGCACCG**

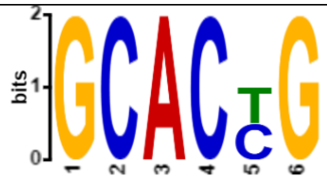	1.5e+003	11.76	**Rv0729c_7** GACTATTGCA GCACCG<br>**Rv0729c_10** GGTGGTGCAT GCACTG GGGCTGGGAG
